# Dietary Sodium Suppresses Digestive Efficiency via the Renin-Angiotensin System

**DOI:** 10.1038/srep11123

**Published:** 2015-06-11

**Authors:** Benjamin J. Weidemann, Susan Voong, Fabiola I. Morales-Santiago, Michael Z. Kahn, Jonathan Ni, Nicole K. Littlejohn, Kristin E. Claflin, Colin M.L. Burnett, Nicole A. Pearson, Michael L. Lutter, Justin L. Grobe

**Affiliations:** 1 Departments of Pharmacology, University of Iowa, Iowa City, IA.; 2 Departments of Psychiatry, University of Iowa, Iowa City, IA.; 3 The Fraternal Order of Eagles’ Diabetes Research Center, University of Iowa, Iowa City, IA.; 4 The Obesity Research and Education Initiative, University of Iowa, Iowa City, IA.; 5 The Center for Hypertension Research, University of Iowa, Iowa City, IA

## Abstract

Dietary fats and sodium are both palatable and are hypothesized to synergistically contribute to ingestive behavior and thereby obesity. Contrary to this hypothesis, C57BL/6J mice fed a 45% high fat diet exhibited weight gain that was inhibited by increased dietary sodium content. This suppressive effect of dietary sodium upon weight gain was mediated specifically through a reduction in digestive efficiency, with no effects on food intake behavior, physical activity, or resting metabolism. Replacement of circulating angiotensin II levels reversed the effects of high dietary sodium to suppress digestive efficiency. While the AT_1_ receptor antagonist losartan had no effect in mice fed low sodium, the AT_2_ receptor antagonist PD-123,319 suppressed digestive efficiency. Correspondingly, genetic deletion of the AT_2_ receptor in FVB/NCrl mice resulted in suppressed digestive efficiency even on a standard chow diet. Together these data underscore the importance of digestive efficiency in the pathogenesis of obesity, and implicate dietary sodium, the renin-angiotensin system, and the AT_2_ receptor in the control of digestive efficiency regardless of mouse strain or macronutrient composition of the diet. These findings highlight the need for greater understanding of nutrient absorption control physiology, and prompt more uniform assessment of digestive efficiency in animal studies of energy balance.

It is generally held that excess consumption of sodium salts and fats lead to poor health outcomes. Excess sodium intake is associated with obesity and the metabolic syndrome^1^, reduced insulin sensitivity^2^, and cardiovascular disease and mortality^3^. Excess fat intake is similarly associated with obesity^4^, and is used to drive animal models of diet-induced obesity^5^. It is important to recognize, however, that considerable debate remains as to a causal role (versus simply a correlation) between these factors and human disease at the population level^6^.

Fast- and processed foods, characterized by high levels of fat and sodium, have been suggested to activate innate mechanisms of reward. It has been posited that these types of food may thus elicit addictive behavior, which may contribute to obesity through excessive caloric intake^7^. Sodium and fat are both palatable to humans and non-human animals, though preferences for one or both can be modulated by various interventions^8^. Together, these findings led us to hypothesize a synergistic effect of dietary fat and sodium to increase food intake and thereby body mass.

The renin-angiotensin system (RAS) is critically involved in cardiovascular physiology, and its role in metabolic physiology is only more recently appreciated^9^. Briefly, obesity is positively correlated with circulating RAS activity in humans and animal models^10^,^11^,^12^,^13^,^14^,^15^,^16^,^17^,^18^,^19^. Genetic or pharmacological interference with the RAS in rodents results in weight loss, reduced adiposity, and/or altered adipose development^20^,^21^,^22^,^23^,^24^,^25^,^26^,^27^,^28^. There is also growing evidence for opposing, tissue/site-specific roles for the RAS in the control of appetite/ingestive behavior^29^,^30^ and in the control of resting metabolism^9^,^31^,^32^,^33^,^34^. As the RAS is strongly suppressed by dietary sodium^35^, this led us to the secondary hypothesis that any observed metabolic consequences of altered dietary sodium may be mediated through its modulation of the RAS.

## Results

### Dietary sodium suppresses HFD-weight gain

**Figure 1:**
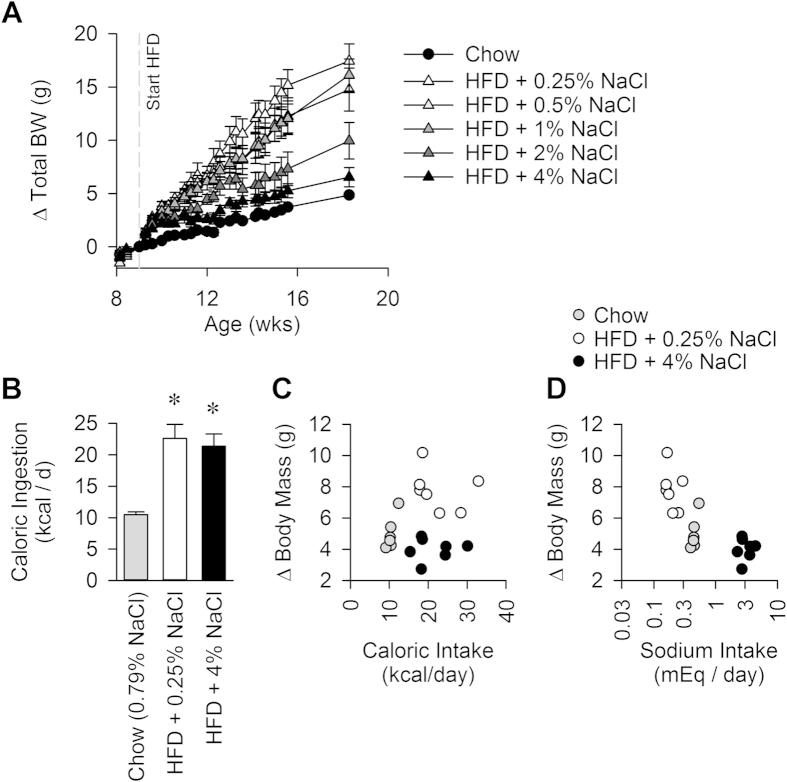
(**A**) Body mass gains over time on various diets. 2-way RM ANOVA: Diet P < 0.001, Time P < 0.001, Diet x Time P < 0.001. N = 5 for each group. (**B**) Daily caloric ingestion during the 5th week of dietary intervention. (**C**) Correlation of body mass gained vs daily caloric intake. (**D**) Correlation of body mass gained vs daily sodium intake. N = 6 chow, 7 HFD + 0.25% NaCl, and 7 HFD + 4% NaCl. (Panels **B**–**D**). *P<0.05 vs Chow.

Placing adult wildtype C57BL/6J mice on a high fat diet (HFD; Table 1) resulted in an immediate and sustained increase in weight gain compared to mice that remained on standard chow. Surprisingly, and contrary to our original hypothesis, dietary sodium caused a dose-dependent *reduction* in weight gain during HFD-feeding (Fig. 1A). This increase in weight gain was primarily due to a robust, early increase in adiposity and slower subsequent (possibly reflexive) increases in lean and fluid masses (Figure S1A). Animals maintained on HFD with lower sodium content exhibited specific expansions of traditional white adipose tissues, including subcutaneous inguinal and perigenital fat pads, while other tissues remained largely unchanged by diet (interscapular “brown” adipose, heart, liver and kidney) (Figure S1B).

### Dietary sodium content did not alter total food intake, despite effects on preference

To assess whether differences in food ingestive behavior could explain the observed effects on weight gain, food intake was measured in mice fed chow, HFD + 0.25% NaCl, and HFD + 4% NaCl for five weeks. Caloric intake (mass of food ingested multiplied by the caloric density) was significantly increased with HFD, but sodium content had no effect upon caloric intake (Fig. 1B). In contrast, the three diets had large, expected effects upon total daily sodium intake (Figure S1C). When the net body mass change over five weeks was plotted against total caloric intake and total sodium intake, no relationship between body mass change and caloric intake was observed (Fig. 1C). In contrast, a significant inverse correlation was observed between body mass gains and sodium ingestion using an inverse 1^st^ order polynomial regression:
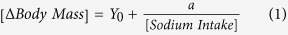
with body mass in grams, and sodium intake in mEq/d; R^2^ = 0.71; Y_0_ = 3.687 ± 0.379, P < 0.0001; a = 0.739 ± 0.111, P < 0.0001 (Fig. 1D). When animals were allowed to choose between pairs of HFD with low (0.25%), medium (1%), or high (4%) NaCl content, it became readily apparent that mice prefer diets with lower sodium content (Figure S1D).

### Dietary sodium content did not change metabolic rate or physical activity

**Figure 2:**
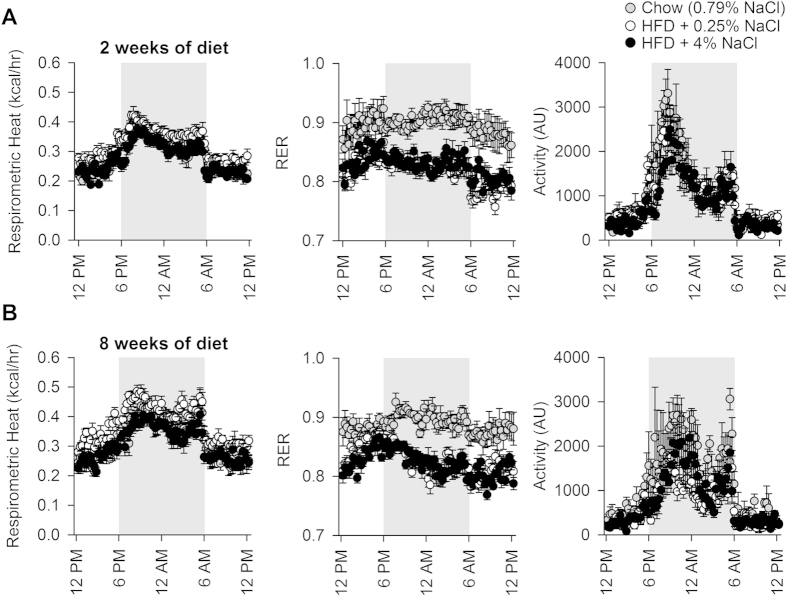
(**A**) Respirometric (aerobic) metabolic rate, respiratory exchange ratio (RER), and spontaneous physical activity of mice with 2 weeks of dietary intervention. (**B**) Respirometric (aerobic) metabolic rate, respiratory exchange ratio (RER), and spontaneous physical activity of mice with 8 weeks of dietary intervention. N = 4 chow, 6 HFD + 0.25% NaCl, and 6 HFD + 4% NaCl.

Metabolic effects of dietary fat and sodium were assessed over the light:dark cycle using an OxyMax system (Columbus Instruments). Aerobic metabolic rate, determined by respirometry, was indistinguishable across groups with two weeks of dietary intervention (Fig. 2A). Importantly, RER was suppressed in both HFD groups, as would be expected with increased metabolic utilization of fatty acids. Spontaneous physical activity was indistinguishable across groups. A similar lack of effect of the dietary interventions was maintained through eight weeks of treatment (Fig. 2B), save a slight increase in metabolic rate in the HFD + 0.25% NaCl group. This divergence is almost certainly due to the increased body mass of the animals after eight weeks on the diet.

Resting metabolic rate (RMR) was specifically examined using a high-resolution combined direct calorimeter/respirometer after 2.5 weeks of dietary intervention. Aerobic RMR, estimated by respirometry, appeared to increase with HFD feeding (Figure S2A). Total RMR as measured by direct calorimetry, however, was unchanged by any dietary interventions (Figure S2B).

### Dietary sodium suppresses circulating RAS activity

Blood pressure was assessed daily in one cohort of mice fed chow, HFD + 0.25% NaCl or HFD + 4% NaCl. No effects of the various diets were observed by tail-cuff plethysmography (Figure S3A-B). Nonetheless, dietary sodium caused the expected reduction in renal renin expression. Compared to HFD + 0.25% NaCl (n = 7; 1.000-fold by Livak 2^-ΔΔCT^ method; ± 1 sem range = 0.817 to 1.225), renin mRNA from whole-kidney extracts was significantly suppressed with HFD + 4% NaCl (n = 8; 0.590-fold; 0.515 to 0.676; P < 0.05). Plasma concentrations of Ang II exhibited expected trends, with HFD + 0.25% NaCl appearing to increase compared to both chow and HFD + 4% NaCl groups, though group differences did not reach statistical significance (Figure S3C). Treating mice with HFD + 0.25% NaCl also caused a significant reduction in mRNA for renin in whole brain homogenate that was largely reversed in mice fed HFD + 4% NaCl (Figure S4A).

### Dietary sodium suppresses digestive efficiency through suppression of the RAS

**Figure 3:**
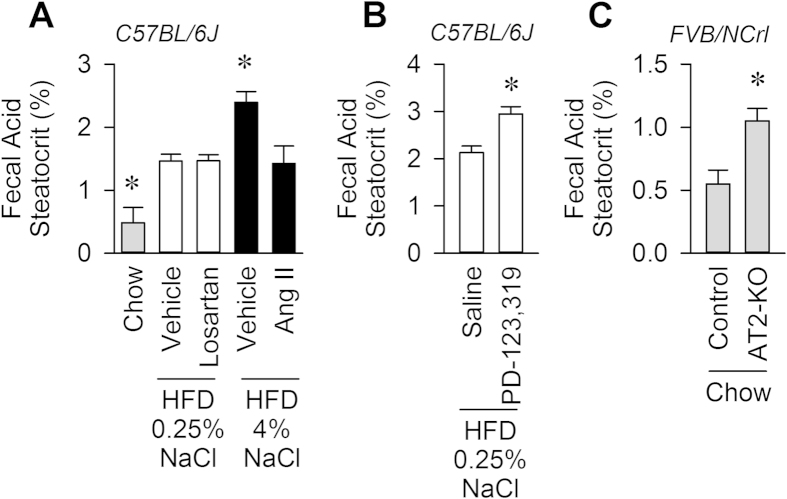
Dietary sodium suppresses digestive efficiency through inhibition of the RAS. (**A**) Loss of fats to stool, assessed by fecal acid steatocrit. N = 7 chow, 8 HFD + 0.25% NaCl, 8 HFD + 0.25% NaCl + the AT_1_ antagonist Losartan (22 μg/hr, s.c.), 8 HFD + 4% NaCl, and 8 HFD + 4% NaCl + angiotensin II (Ang II, 100 ng/kg/min, s.c.). (**B**) Digestive efficiency assessed by fecal acid steatocrit, in C57BL/6J mice chronically infused for 4 weeks with saline vehicle (n = 8) or the AT_2_ antagonist, PD-123,319 (0.55 μg/hr, n = 10) while maintained on HFD + 0.25% NaCl diet. (**C**) Digestive efficiency assessed by fecal acid steatocrit in mice harboring a null allele of the AT_2_ receptor (AT2-KO, n = 6) and littermate controls (n = 6), all maintained on the FVB/NCrl background strain and fed standard chow. *P < 0.05 versus all other groups by Tukey multiple-comparisons procedure (panel **A**) or by independent t-test (panels **B**, **C**).

Digestive efficiency, as assessed by fecal acid steatocrit, was reduced by increased dietary sodium. Mice fed HFD + 4% NaCl lost roughly double the amount of fatty acid to the stool as mice fed HFD + 0.25% NaCl (Fig. 3A). Chronic infusion of a non-pressor dose of Ang II (100 ng/kg/min, s.c.) into mice fed HFD + 4% NaCl increased digestive efficiency to the level of animals fed HFD + 0.25% NaCl. Interestingly, chronic infusion of the Ang II AT_1_ receptor antagonist losartan (22 μg/hr, s.c.) had no effect to reduce digestive efficiency in mice fed HFD + 0.25% NaCl.

Examination of RAS gene expression patterns throughout the intestinal tract confirmed expression of renin, angiotensinogen, angiotensin converting enzyme (ACE), and (ACE)-2, AT_1A_ and AT_2_ receptor expression throughout the duodenum, jejunum, ileum and cecum (Figure S4B). HFD, regardless of sodium content, had a suppressive effect on ACE2 in duodenum and jejunum; and AT_1A_ and AT_2_ appear to exhibit an moderate suppression in ileum with low sodium diet or angiotensin II infusion.

### The angiotensin AT_2_ receptor reduces digestive efficiency

In contrast to the lack of effect of chronic infusion of the AT_1_ receptor antagonist, chronic infusion of the AT_2_ receptor antagonist, PD-123,319 (0.55 μg/hr, s.c.) into wildtype C57BL/6J mice fed HFD + 0.25% NaCl caused a significant suppression of digestive efficiency, compared to mice chronically infused with saline vehicle (Fig. 3B).

Finally, to overcome the shortcomings of selectivity posed by studies utilizing pharmacological antagonists, digestive efficiency was also assessed in mice carrying a null allele for the AT_2_ receptor (AT2-KO), maintained on the FVB/NCrl background strain. AT2-KO mice maintained on standard chow (13.7 ± 0.7 weeks of age, 26.91 ± 1.20 grams, n = 6) exhibited a significantly suppressed digestive efficiency compared to littermate controls (13.9 ± 0.5 weeks of age, 26.88 ± 0.82 grams, n = 6), when assessed using fecal acid steatocrit (Fig. 3C).

## Discussion

**Figure 4:**
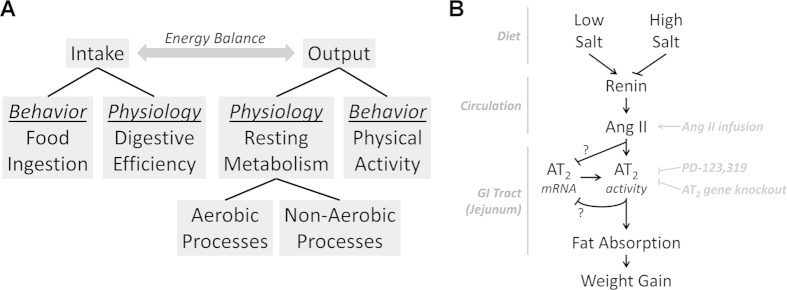
(**A**) Major biological and behavioral contributors to energy balance. Dietary sodium prevents weight gain during high-fat feeding specifically through suppression of digestive efficiency, without effect on food intake, resting metabolic processes, or physical activity. (**B**) Hypothesized mechanism by which dietary sodium modulates digestive efficiency, and subsequently weight gain, through suppression of the circulating RAS and thereby AT_2_ receptor activation.

The current study demonstrates that dietary sodium content can suppress weight gain during HFD feeding. This appears to be mediated exclusively through a suppression of digestive efficiency, which results from a sodium-induced suppression of the RAS. Interestingly the AT_2_ receptor, not AT_1_ receptor, was implicated in the control of digestive efficiency. Of note, the effect of AT_2_ modulation on digestive efficiency was consistently observed in both C57BL/6J and FVB/NCrl strains of mice, maintained on either a custom-formulated 45% high fat diet or on a standard chow with vastly different compositions and obtained from different vendors. Collectively, these results implicate dietary sodium and the RAS in the control of digestive efficiency, and underscore the physiological relevance of digestive efficiency in the pathogenesis of diet-induced obesity (Fig. 4).

In 1960, Dahl demonstrated that dietary sodium intakes across populations positively correlated with the incidence of hypertension^36^. Specifically, NaCl consumption varied from roughly 5 g/d in Alaskan Eskimos where hypertension was extremely rare, to 25 g/d in Northern Japanese, where nearly one third of the population exhibited hypertension. Estimating an adult human’s daily food intake at 1.2 kg/d^37^, this would support a range of 0.4–2.1% NaCl in typical human foods. Similarly, the INTERSALT study documented a range of 23 mg/d sodium intake in the Yanomamo tribe of Brazil, to 6 g/d in China^38^,^39^. Again using an average 1.2 kg/d food ingestion and assuming all of the sodium was consumed as NaCl, this would equate to a range of 0.005–1.275% NaCl in food. Similarly, O’Donnell recently reported on behalf of the PURE study (including 101,945 individual human subjects across 17 countries) that the average sodium intake is 4.93 g/d, which would equate to 12.53 g/d of NaCl; using similar food intake estimates as above, this yields a 1.04% NaCl diet equivalent^40^. According to nutritional information provided by the McDonald’s USA corporation, a Quarter Pounder with Cheese and medium-sized French Fries meal includes the rough equivalent of 1.1% NaCl^41^. Thus, our examination of the effects of 0.25–4% NaCl in the context of a high fat diet is relevant and applicable to humans across a wide spectrum of cultures and populations.

Digestive efficiency is a relatively safe and moderately efficacious target for the treatment of obesity. Orlistat, also known as Xenical or tetrahydrolipstatin, is a pancreatic lipase inhibitor that was approved for human use by the United States Food & Drug Administration in 1999. Given its safe use and extremely low potential for addiction or abuse, it was approved for over-the-counter sale as “Alli” in 2007. This compound effectively inhibits the enzymatic digestion of dietary fats and thereby prevents the absorption of a fraction of ingested lipids by the gastrointestinal tract. Orlistat appears to maximally inhibit the absorption of roughly one third of ingested lipids^42^,^43^. It is unclear whether targeting the secretion of pancreatic enzymes may have a greater maximal efficacy than competitively inhibiting these enzymes. A greater understanding of the mechanisms controlling digestive efficiency is needed before new therapeutic targets can be identified.

The renin-angiotensin system, or its downstream mediators, may represent novel targets for the modulation of digestive efficiency. As recently reviewed by Garg *et al.*^44^, essentially all components of the RAS are expressed throughout the digestive system. Renin is readily present in the circulation and its mRNA has been detected in human small intestine^45^ and colon^46^. ACE and ACE2 are strongly expressed in the small intestine (*in fact, the highest human tissue concentrations of these enzymes are in intestine*)^47^,^48^,^49^,^50^ and colon^46^, and neutral endopeptidase (neprilysin) shows similar patterns of expression^51^. AT_1_ receptors are localized to the epithelial brush border, circular and longitudinal muscle layers and myenteric plexus of the small intestine, but AT_2_ receptors are restricted to the myenteric plexus^52^,^53^,^54^. AT_1_ and AT_2_ are also expressed in various layers of the colon^46^, stomach^55^, and esophagus^56^,^57^. Angiotensinogen is expressed throughout the small intestine^58^, colon^59^, and stomach^55^. Various RAS peptides including angiotensin (Ang) I, Ang II, and Ang-(1-7) have been documented in various portions of the GI tract^52^,^60^. Thus, the RAS is present in the viscera and it is therefore positioned to potentially play an important role in the control of digestive function.

Ang II action at AT_2_ receptors has been documented to functionally influence digestive system function. Ang II stimulates apoptosis in intestinal epithelial cells via the AT_2_ receptor^61^. Activation of AT_2_ by CGP-42112a causes increased nitric oxide production in jejunal mucosa^62^. Low-dose Ang II delivery stimulates jejunal water and electrolyte absorption, and this effect appears to be mediated through an AT_2_-cGMP signaling mechanism^63^. While AT_1_ and AT_2_ receptors are both present on pancreatic acinar cells and secretion of digestive enzymes by these cells is stimulated by Ang II, this effect is blocked by losartan but not PD-123,319, highlighting a role for AT_1_ but not AT_2_^64^. The RAS may therefore mediate its effects on caloric absorption through a complex modulation of digestive enzyme secretion and gastric motility, or other as-yet unidentified mechanisms.

Takahashi previously demonstrated that genetic disruption of the renin gene in mice resulted in protection from HFD-induced weight gain, that this mechanism involved both an increase in thermogenesis and also a reduction in digestive efficiency, and that the metabolic consequences of renin deficiency could be rescued by Ang II replacement^20^. While various genetic and pharmacological manipulations to other components of the RAS have anti-obesity effects in rodents, we have been unable to find any mention of effects of these manipulations upon digestive efficiency. We hypothesize that this may simply be a consequence of many laboratories ignoring the physiological significance of digestive efficiency modulation, as food intake data are very rarely corrected for digestive efficiency in discussions of the relative effects of genetic, pharmacological, and dietary manipulations upon total energy balance.

Unlike Takahashi’s studies of global renin knockout mice^20^, in the present study we did not observe changes in net thermogenesis/energy expenditure. This may reflect the *universal* disruption of renin in genetic knockout mice, versus a modulation of predominantly the *circulating* RAS with dietary sodium changes, as salt-sensitive models of hypertension exhibit suppressed circulating RAS activity but elevated brain RAS activity^65^,^66^,^67^. It appears, however, that dietary fats and sodium content may shift the form of energy utilization toward aerobic processes and away from non-aerobic processes. Respirometric estimates of heat production ignore the contributions of anaerobic and nitrogen metabolisms, and are subject to a long list of required assumptions regarding the test subject’s physiology^68^. It has been demonstrated that respirometry grossly underestimates true total metabolic rate by up to 38% in various endothermic species^69^. We previously demonstrated that resting metabolic rate (RMR) of laboratory mice is underestimated by respirometric methods, and that this rate of underestimation is highly variable^31^. The variability of underestimation is so great that qualitatively false negative and false positive conclusions regarding the effects of pharmacological and genetic manipulations in mice can result^31^. Very recently we demonstrated that respirometry indicates a false positive effect of switching from standard chow to a 45% HFD upon total RMR in wildtype C57BL/6J mice^70^. Specifically, we determined that with a shift from chow to HFD, respirometry detects a significant (~0.01 kcal/hr) increase in aerobic RMR but fails to capture the simultaneous and equally large decrease in non-aerobic RMR. That observation is now replicated in the present study, where animals switched to HFD exhibited an apparent increase in aerobic RMR but no change in total RMR. In other words, dietary shifts qualitatively change the contributions of various types of resting metabolic processes (i.e. – aerobic vs non-aerobic), but have no observed quantitative effect on total resting energy expenditure.

In summary, dietary sodium causes a dose-dependent suppression of digestive efficiency through inhibition of the RAS, and this effect is sufficient to completely prevent HFD-induced weight gain. Given the importance of digestive efficiency to health and disease, and the proven utility of targeting digestive efficiency to treat obesity (e.g. – orlistat), we hypothesize that further investigations into mechanisms (such as the RAS and its AT_2_ receptor) that control digestive efficiency may lead to the development of more efficacious anti-obesity therapeutics.

## Methods

### Animals

Wildtype male C57BL/6J mice were obtained from the Jackson Laboratories at 5-6 weeks of age. Mice were acclimated to housing conditions (23 °C, 12:12 light:dark cycle) for several weeks before studies were initiated at 9 weeks of age. AT_2_ receptor-deficient mice and littermate controls maintained on the FVB/NCrl background strain were obtained from an in-house breeding colony, originally obtained from Drs. Victor J. Dzau and Richard E. Pratt (Duke University). Animals were allowed ad libitum access to water and various diets throughout all studies except for acute measurements as described below. Selected mice underwent subcutaneous implantation of osmotic minipumps under isoflurane anesthesia to deliver a non-pressor dose of angiotensin II (Ang II; 100 ng/kg/min), the Ang II type 1 receptor antagonist, losartan (22 ug/hr), or the Ang II type 2 receptor antagonist, PD-123,319 (0.55 μg/hr). All animal experiments were approved by the University of Iowa Institutional Animal Care and Use Committee, and all methods were in accordance with the Guide for the Care and Use of Laboratory Animals^71^.

### Diets

Mice were maintained on a standard rodent chow (Teklad 7013) unless otherwise stated. Custom high-fat diets (BioServ) were developed to provide a high fat content (45% of kcal from fat) with varied concentrations of NaCl (0.25% to 4%). Compositions of all diets are described in Table 1.

### Body Composition

Body composition was assessed by nuclear magnetic resonance (NMR; Bruker LF90II)^9^,^70^. Briefly, awake/unanesthetized animals were placed into a polycarbonate restraint tube and placed inside the scanner for roughly 1 minute to perform the analyses. Animals were immediately returned to their home cage.

### Food Intake and Preference

Food intake was assessed in home cages under standard housing conditions. Food preference assessments were also performed in home cages, with two food options presented simultaneously in identical hoppers on opposite ends of the cage. Intake of each food was assessed daily for one week and averaged within animal before making group comparisons.

### Total Calorimetry/Resting Metabolic Rates

Aerobic and total (i.e. – aerobic + anaerobic + nitrogenous) resting metabolic rates (RMR) were assessed simultaneously using a combined or “total” calorimeter system, as recently described in detail^31^,^70^. This system consists of a custom-fabricated gradient-layer direct calorimeter (to directly measure total heat dissipation and retention by the mouse) and a conventional push-pull respirometry system (to estimate aerobic RMR using empirically derived equations, as below). Animals were individually tested by placing a single mouse in the chamber at 8 AM (2 hours into the light phase) and continuously monitoring total heat dissipation and gas exchange for roughly six hours. Total and aerobic RMR were calculated when the animal was stably asleep in the chamber. RER was calculated as the ratio of CO_2_ produced (VCO_2_) versus O_2_ consumed (VO_2_). Aerobic heat (in kcal/hr) was calculated using the equation derived from Lusk^72^, with STP-corrected gas consumption/production rates (in mL/min)^31^,^70^.
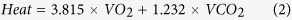


### OxyMax/Comprehensive Laboratory Animal Monitoring System (CLAMS)

Respirometric heat production, respiratory exchange ratio (RER), physical activity by photoelectric beam break, and food intake/patterning were assessed using a 16-chamber OxyMax CLAMS system (Columbus Instruments). Animals were housed individually in specialized OxyMax home cages for four nights in total. Ambient temperature was maintained at 30 ± 1 °C throughout the testing period. Animals were acclimated to the system for 48 hours, and data were recorded and analyzed from the remaining 48 hours of the testing period. Data from 17 minute bins were averaged across days within animal before group comparisons were made. Aerobic heat was calculated by the system using the Lusk equation, as above^72^.

### Blood Pressure

Blood pressure was assessed by tail-cuff plethysmography (Visitech). Testing was performed daily five days per week, and involved restraining the animals on a warmed heating surface. Animals were instrumented with a pneumatic occlusion cuff proximal to an infrared pulse oximeter. Flow was occluded by pressurizing the cuff, and the pressure at the loss of pulse detection was noted. Thirty occlusion cycles were performed over thirty minutes, once daily. Data from the first week of testing were discarded as training; remaining data were averaged within animal, within day, and then within week before group comparisons were made.

### RAS component mRNA

Animals were sacrificed by CO_2_ asphyxiation. Kidneys, brain, and intestinal sections were removed by blunt dissection and frozen on dry ice. Tissue was later thawed and RNA was isolated by trizol followed by use of PureLink RNA kits (Ambion). RNA was then examined for specific gene content by realtime RT-PCR (SYBR green method) as previously^34^,^73^, using primer pairs previously reported^74^. Importantly, as two distinct isoforms of renin are expressed in the brain^75^, we used a primer set for renin (Forward 5’-TGAAGAAGGCTGTGCGGTAGT-3’, and Reverse 5’-TCCCAGGTCAAAGGAAATGTC-3’) which spans exons 7 and 8 of the renin locus, and thereby detects mRNA for both intracellular and secreted isoforms of the enzyme. Fold changes in gene expression were calculated using the Livak 2^-ΔΔCt^ method^76^, and analytical statistics were performed on ΔCt values.

### Circulating Angiotensin II (and III)

Trunk blood was collected at sacrifice into tubes coated with EDTA, and plasma was isolated and frozen at −80 °C after centrifugation for 5 minutes at 5,000 × g. Plasma was subsequently thawed and then extracted using a commercially-available C18 SEP-Column kit per manufacturer’s instructions (Peninsula Laboratories International, Inc.; Catalog Number S-5000). 100 uL of plasma was extracted, lyophilized, and stored at −80 °C. The dried extract was then reconstituted in 150 uL of EIA buffer and analyzed using a commercially-available EIA kit per manufacturer’s instructions (Peninsula Laboratories International, Inc.; Catalog Number S-1133).

### Fecal Acid Steatocrit

Mice were individually placed into glass beakers at 10 AM (4 hours into the light phase), and fresh fecal pellets were collected until eight pellets were collected per animal^20^. 50 ± 1 mg of fresh fecal material was then isolated from each animal. This 50 mg sample was then pulverized and dissolved in 200 μL of freshly prepared 1 N perchloric acid. Next, 100 μL of 0.5% Oil-Red-O was added to the mixture. The slurry was then loaded into non-heparinized micro-hematocrit tubes and spun for 10 minutes at 10,000 × g. Tubes were then aligned and digitally photographed. The proportion of red-stained oil layer to the total sample length was calculated using ImageJ software from the NIH.

### Statistics

Throughout, ANOVA-based analyses (with repeated measures as appropriate) and independent t-test were utilized. Tukey multiple-comparisons procedures were used for post-hoc analyses. Differences were considered significant at P < 0.05. Data are reported as mean ± 1 sem, throughout.

## Additional Information

**How to cite this article**: Weidemann, B. J. *et al.* Dietary Sodium Suppresses Digestive Efficiency via the Renin-Angiotensin System. *Sci. Rep.* **5**, 11123; doi: 10.1038/srep11123 (2015).

## Supplementary Material

Supplementary Information

## Figures and Tables

**Table 1:** **Compositions of Diets.**

**Diet**	**Vendor, Stock**	**Fat (kcal %)**	**NaCl (%)**	**Na (%)**	**Na (mEq/g)**	**Caloric Density (kcal/g)**
Chow	Teklad 7013	18	0.79	0.31	0.135	3.13
HFD + 0.25% NaCl	BioServ F6519	45	0.25	0.10	0.043	4.86
HFD + 0.5% NaCl	BioServ F6520	45	0.50	0.20	0.086	4.86
HFD + 1% NaCl	BioServ F6521	45	1.00	0.38	0.167	4.82
HFD + 2% NaCl	BioServ F6522	45	2.00	0.79	0.342	4.77
HFD + 4% NaCl	BioServ F6523	45	4.00	1.57	0.684	4.67
