# Supplementary material for: Dietary Sodium Suppresses Digestive Efficiency via the Renin-Angiotensin System
Source: Sci Rep. 2015 Jun 11;5:11123. doi: 10.1038/srep11123 (PMC4464075; doi:10.1038/srep11123)
Supplement: Supplementary Information [file srep11123-s1.pdf]

SUPPLEMENTAL DATA

Dietary Sodium Suppresses Digestive Efficiency via the Renin-Angiotensin System

Benjamin J. Weidemann[1], Susan Voong[1], Fabiola I. Morales-Santiago[1], Michael Z. Kahn[2], Jonathan Ni[1], Nicole K. Littlejohn[1], Kristin E. Claflin[1], Colin M.L. Burnett[1], Nicole A. Pearson[1], Michael L. Lutter[2,3,4], and Justin L. Grobe[1,3,4,5]

Departments of [1]Pharmacology and [2]Psychiatry, [3]the Fraternal Order of Eagles' Diabetes Research Center, [4]the Obesity Research and Education Initiative, and [5]the Center for Hypertension Research, University of Iowa, Iowa City, IA

Short Title: Sodium, Angiotensin, and Digestive Efficiency

Corresponding Author:
Justin L. Grobe, PhD, FAHA
Department of Pharmacology, University of Iowa
51 Newton Rd., 2-307 BSB
Iowa City, IA 52242
Tel: (319) 353-5789
Fax: (319) 335-8930
Email: justin-grobe@uiowa.edu

Figure S1. (A) Body composition over time on various diets. Lean: Diet P=0.053, Time P<0.001, Diet x Time P=0.063. Fat: Diet P=0.003, Time P<0.001, Diet x Time P<0.001. Fluid: Diet P<0.001, Time P<0.001, Diet x Time P=0.002. N=5 mice / group for all endpoints. (B) Tissue masses at sacrifice. N=5 mice / group for all endpoints. (C) Daily sodium ingestion during the 5th week of dietary intervention. (D) Relative intake of diets when presented as paired choice tests. *P<0.05 vs chow by Tukey multiple-comparisons procedure following P<0.05 one-way ANOVA result (panels A, B) or independent t-test (panel D).

Figure S2. (A) Respirometric (aerobic) resting metabolic rate (RMR) with 2.5 weeks of dietary intervention. (B) Total RMR measured by direct calorimetry simultaneously with data in panel A. (C) Respirometric RMR versus total RMR; data from panels A + B. N=5 mice / group for all groups. *P<0.05 vs chow by Tukey multiple-comparisons procedure following P<0.05 one-way ANOVA result.

Figure S3. (A) Systolic blood pressure as determined by tail-cuff plethysmography. (B) Heart rate determined simultaneously with data in panel A. N=6 chow, 7 HFD+0.25% NaCl, and 7 HFD+4% NaCl. (C) Plasma concentration of angiotensin II. N=7 chow, 8 HFD+0.25% NaCl, and 8 HFD+4% NaCl.

Figure S4. (A) mRNA for RAS components in whole-brain homogenate, as determined using realtime RT-PCR. Chow N=7, HFD+0.25% NaCl N=4, HFD+4% NaCl N=5. (B) mRNA for RAS components in segments of the gastrointestinal tract. Duodenum; N=4

for all groups. Jejunum; Chow N=7, HFD+0.25% NaCl N=6, HFD+4% NaCl N=8, HFD+4% NaCl+Ang II N=8. Ileum; Chow N=7, HFD+0.25% NaCl N=8, HFD+4% NaCl N=8, HFD+4% NaCl+Ang II N=8. Cecum; Chow N=6, HFD+0.25% NaCl N=5, HFD+4% NaCl N=7, HFD+4% NaCl+Ang II N=5. *P<0.05 for indicated comparisons, by Tukey multiple-comparisons procedure following P<0.05 one-way ANOVA result.

Figure S1

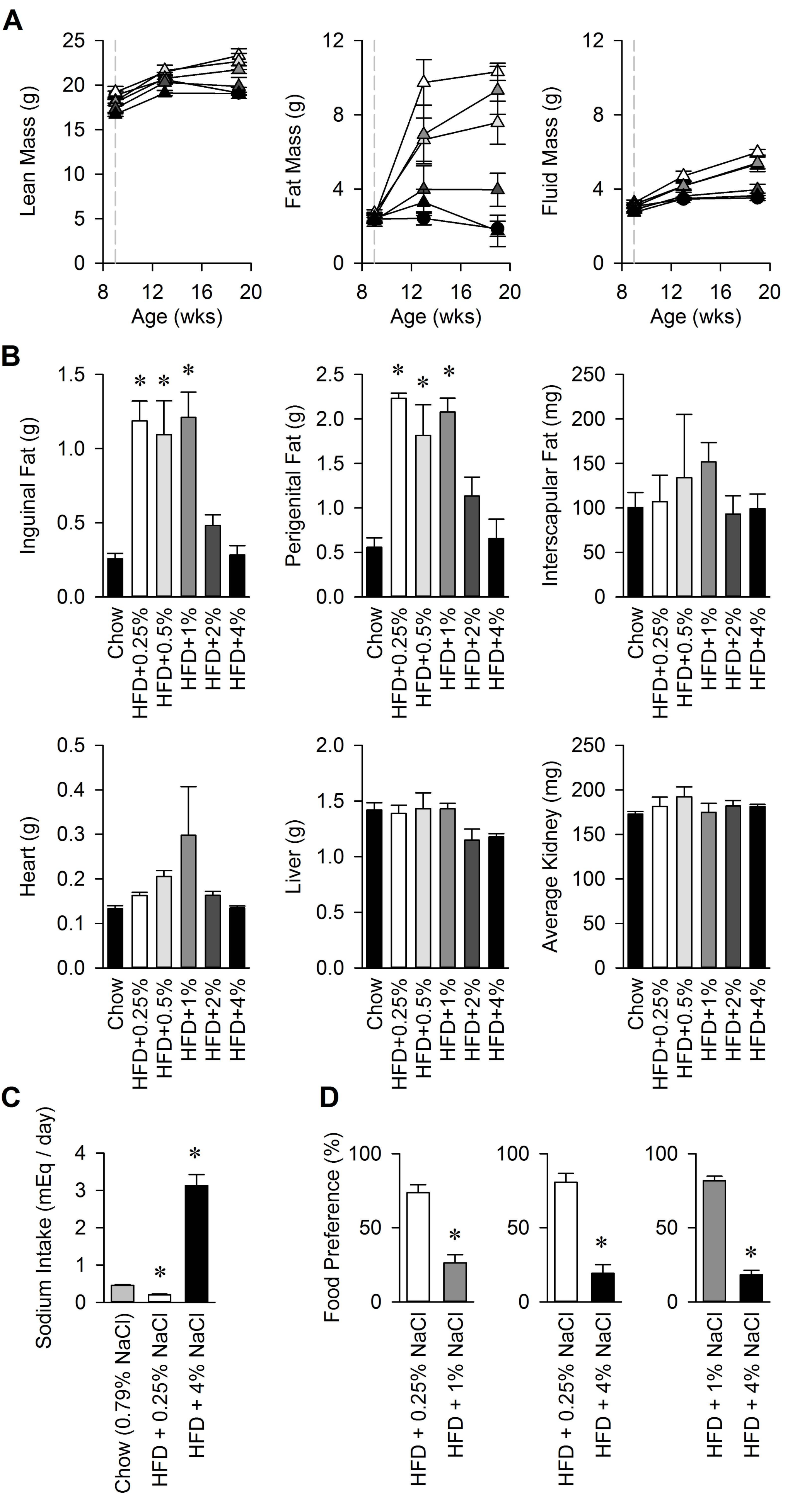
A
Lean Mass (g)
Age (wks)
Fat Mass (g)
Age (wks)
Fluid Mass (g)
Age (wks)
B
Inguinal Fat (g)
Perigenital Fat (g)
Interscapular Fat (mg)
Heart (g)
Liver (g)
Average Kidney (mg)
Chow
HFD+0.25%
HFD+0.5%
HFD+1%
HFD+2%
HFD+4%
C
Sodium Intake (mEq / day)
Chow (0.79% NaCl)
HFD + 0.25% NaCl
HFD + 4% NaCl
D
Food Preference (%)
HFD + 0.25% NaCl
HFD + 1% NaCl
HFD + 0.25% NaCl
HFD + 4% NaCl
HFD + 1% NaCl
HFD + 4% NaCl

Figure S2

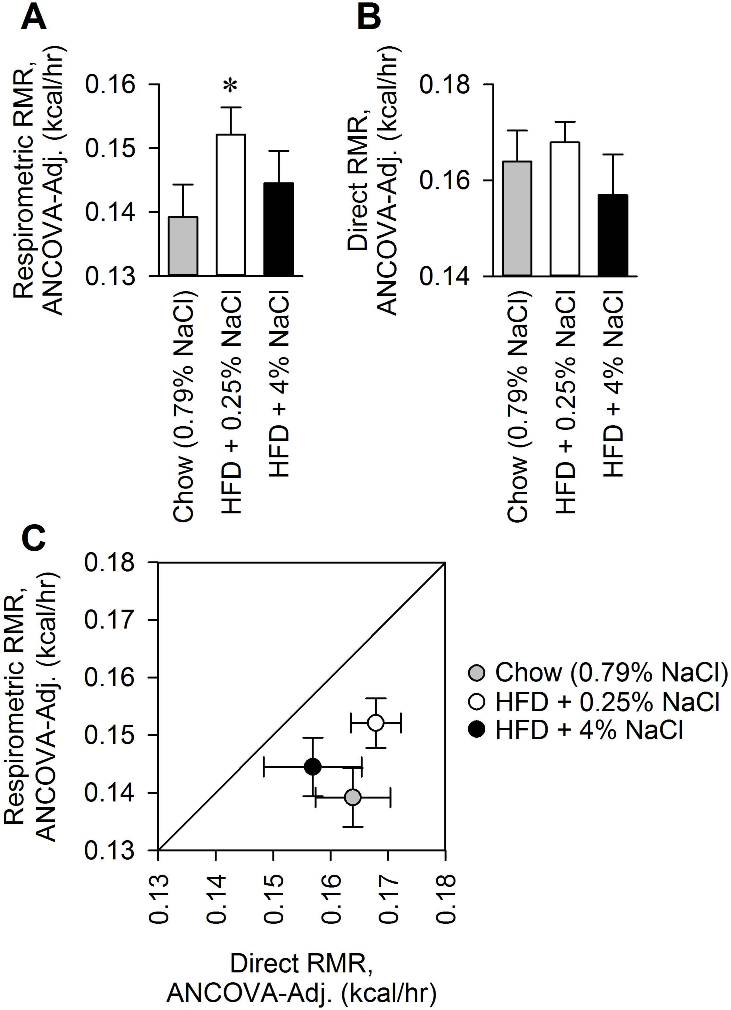

A
Respirometric RMR, ANCOVA-Adj. (kcal/hr)
0.16
0.15
0.14
0.13
*
Chow (0.79% NaCl)
HFD + 0.25% NaCl
HFD + 4% NaCl
B
Direct RMR, ANCOVA-Adj. (kcal/hr)
0.18
0.16
0.14
Chow (0.79% NaCl)
HFD + 0.25% NaCl
HFD + 4% NaCl
C
Respirometric RMR, ANCOVA-Adj. (kcal/hr)
0.18
0.17
0.16
0.15
0.14
0.13
0.13
0.14
0.15
0.16
0.17
0.18
Direct RMR, ANCOVA-Adj. (kcal/hr)
Chow (0.79% NaCl)
HFD + 0.25% NaCl
HFD + 4% NaCl

Figure S3

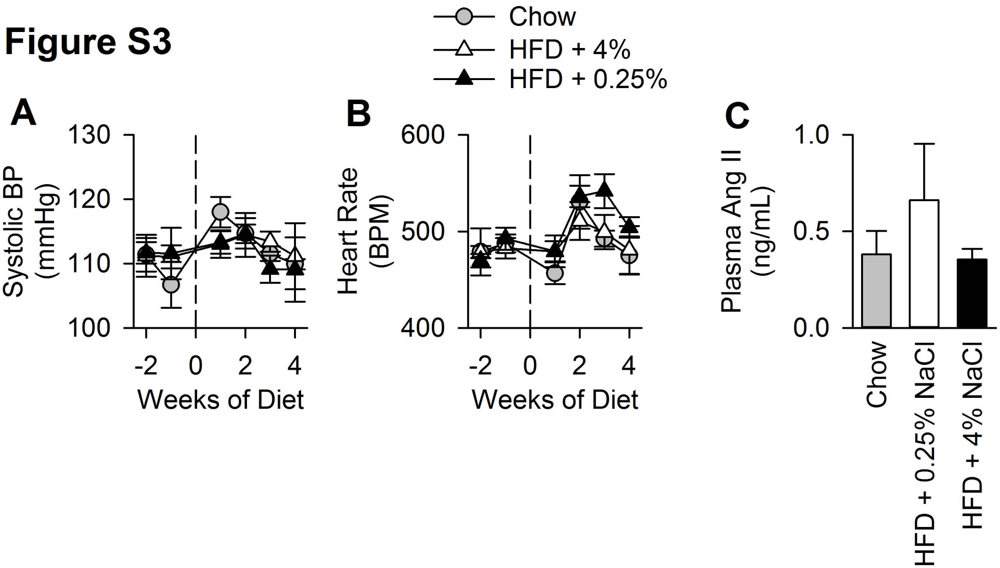
Chow
HFD + 4%
HFD + 0.25%
A
Systolic BP (mmHg)
130
120
110
100
-2
0
2
4
Weeks of Diet
B
Heart Rate (BPM)
600
500
400
-2
0
2
4
Weeks of Diet
C
Plasma Ang II (ng/mL)
1.0
0.5
0.0
Chow
HFD + 0.25% NaCl
HFD + 4% NaCl

Figure S4

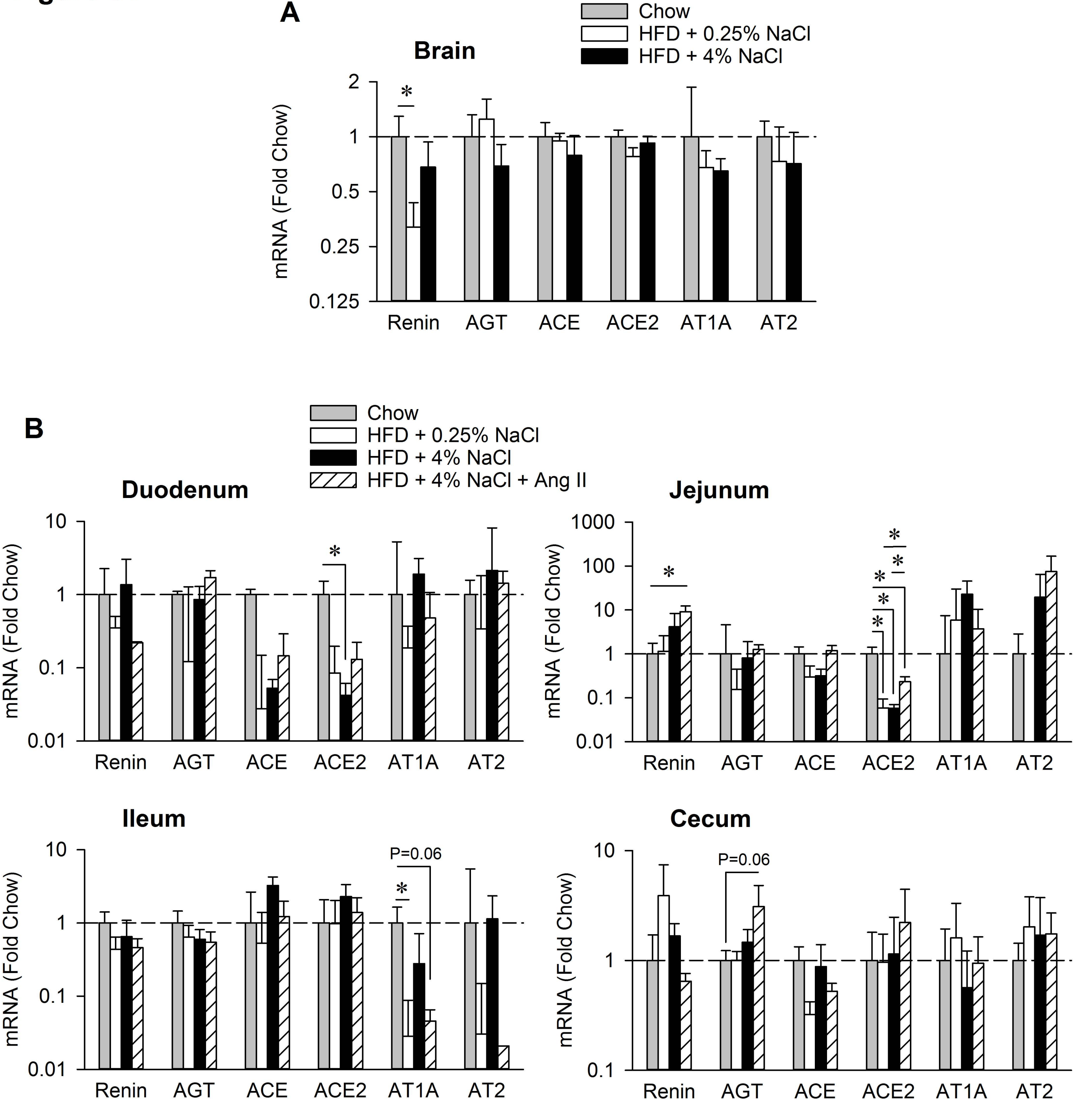
A
Chow
HFD + 0.25% NaCl
HFD + 4% NaCl
Brain
mRNA (Fold Chow)
2
1
0.5
0.25
0.125
*
Renin
AGT
ACE
ACE2
AT1A
AT2
B
Chow
HFD + 0.25% NaCl
HFD + 4% NaCl
HFD + 4% NaCl + Ang II
Duodenum
Jejunum
Ileum
Cecum
mRNA (Fold Chow)
10
1
0.1
0.01
1000
100
P=0.06
Renin
AGT
ACE
ACE2
AT1A
AT2
